# L1TD1 Is a Marker for Undifferentiated Human Embryonic Stem Cells

**DOI:** 10.1371/journal.pone.0019355

**Published:** 2011-04-29

**Authors:** Raymond Ching-Bong Wong, Abel Ibrahim, Helen Fong, Noelle Thompson, Leslie F. Lock, Peter J. Donovan

**Affiliations:** 1 Department of Biological Chemistry, University of California Irvine, Irvine, California, United States of America; 2 Department of Developmental and Cell Biology, University of California Irvine, Irvine, California, United States of America; 3 Sue and Bill Gross Stem Cell Research Centre, University of California Irvine, Irvine, California, United States of America; University of São Paulo, Brazil

## Abstract

**Background:**

Human embryonic stem cells (hESC) are stem cells capable of differentiating into cells representative of the three primary embryonic germ layers. There has been considerable interest in understanding the mechanisms regulating stem cell pluripotency, which will ultimately lead to development of more efficient methods to derive and culture hESC. In particular, Oct4, Sox2 and Nanog are transcription factors known to be important in maintenance of hESC. However, many of the downstream targets of these transcription factors are not well characterized. Furthermore, it remains unknown whether additional novel stem cell factors are involved in the establishment and maintenance of the stem cell state.

**Methodology/Principal Findings:**

Here we show that a novel gene, L1TD1 (also known as FLJ10884 or ECAT11), is abundantly expressed in undifferentiated hESC. Differentiation of hESC via embryoid body (EB) formation or BMP4 treatment results in the rapid down-regulation of L1TD1 expression. Furthermore, populations of undifferentiated and differentiated hESC were sorted using the stem cell markers SSEA4 and TRA160. Our results show that L1TD1 is enriched in the SSEA4-positive or TRA160-positive population of hESC. Using chromatin immunoprecipitation we found enriched association of Nanog to the predicted promoter region of L1TD1. Furthermore, siRNA-mediated knockdown of Nanog in hESC also resulted in downregulation of L1TD1 expression. Finally, using luciferase reporter assay we demonstrated that Nanog can activate the L1TD1 upstream promoter region. Altogether, these results provide evidence that L1TD1 is a downstream target of Nanog.

**Conclusion/Significance:**

Taken together, our results suggest that L1TD1 is a downstream target of Nanog and represents a useful marker for identifying undifferentiated hESC.

## Introduction

Human embryonic stem cells (hESC) are pluripotent cells that can self-renew indefinitely and also generate cells representative of the three primary embryonic germ layers [1], [2]. The latter ability, termed pluripotency, makes hESC an ideal tool to develop cell replacement therapies. However, before the therapeutic potential of hESC can be fully realized, development of a culture system that enhances the production of undifferentiated hESC will be essential. Studies of the molecular mechanisms regulating hESC pluripotency could be helpful in this regard. Several transcription factors are known to be important regulators of pluripotency and self-renewal in hESC, including Oct4, Sox2 and Nanog [3], [4], [5], [6]. These three transcription factors are able to regulate the expression of each other, effectively forming a core transcriptional network governing pluripotency of hESC [7]. A similar transcriptional network between Tcl1, Tbx3 and Esrrb also exist in mouse embryonic stem cells (mESC) [8]. However, whether there are other factors involved in regulating hESC pluripotency remains to be determined. Studies that address these questions could provide critical insights into the mechanisms regulating self-renewal and early differentiation of hESC.

We are interested in the novel gene FLJ10884, which was recently renamed as LINE 1 type transposase domain containing 1 (L1TD1) in the NCBI database (http://www.ncbi.nlm.nih.gov). Moreover, it was categorized as ECAT11, a member of the Embryonic Stem Cell Associated Transcripts (ECAT). The ECAT genes are a set of genes that were found to be enriched in mESC compared to somatic cells using a digital differential display method [9]. Notably, many ECAT genes have been found to play an important role in stem cell biology. For instance, ECAT4 was identified to be Nanog, a master regulator for the maintenance of mESC and hESC [3], [9]. ECAT5 was identified to be Embryonic stem cell expressed Ras (ERas), a Ras-like oncogene that is important in regulating mESC proliferation [10]. ECAT9 was identified as Growth and differentiation factor 3 (GDF3), an important factor that helps maintain mESC pluripotency by inhibiting bone morphogenetic protein (BMP) signaling [11].

In this study, we have characterized the expression of L1TD1 in hESC. We showed that L1TD1 is highly enriched in undifferentiated hESC compared to their differentiated derivatives, indicating that it may be a useful marker to identify undifferentiated hESC. Moreover, we demonstrated that L1TD1 is a target gene of Nanog.

## Results

### L1TD1 is a marker for undifferentiated hESC

**Figure 1:**
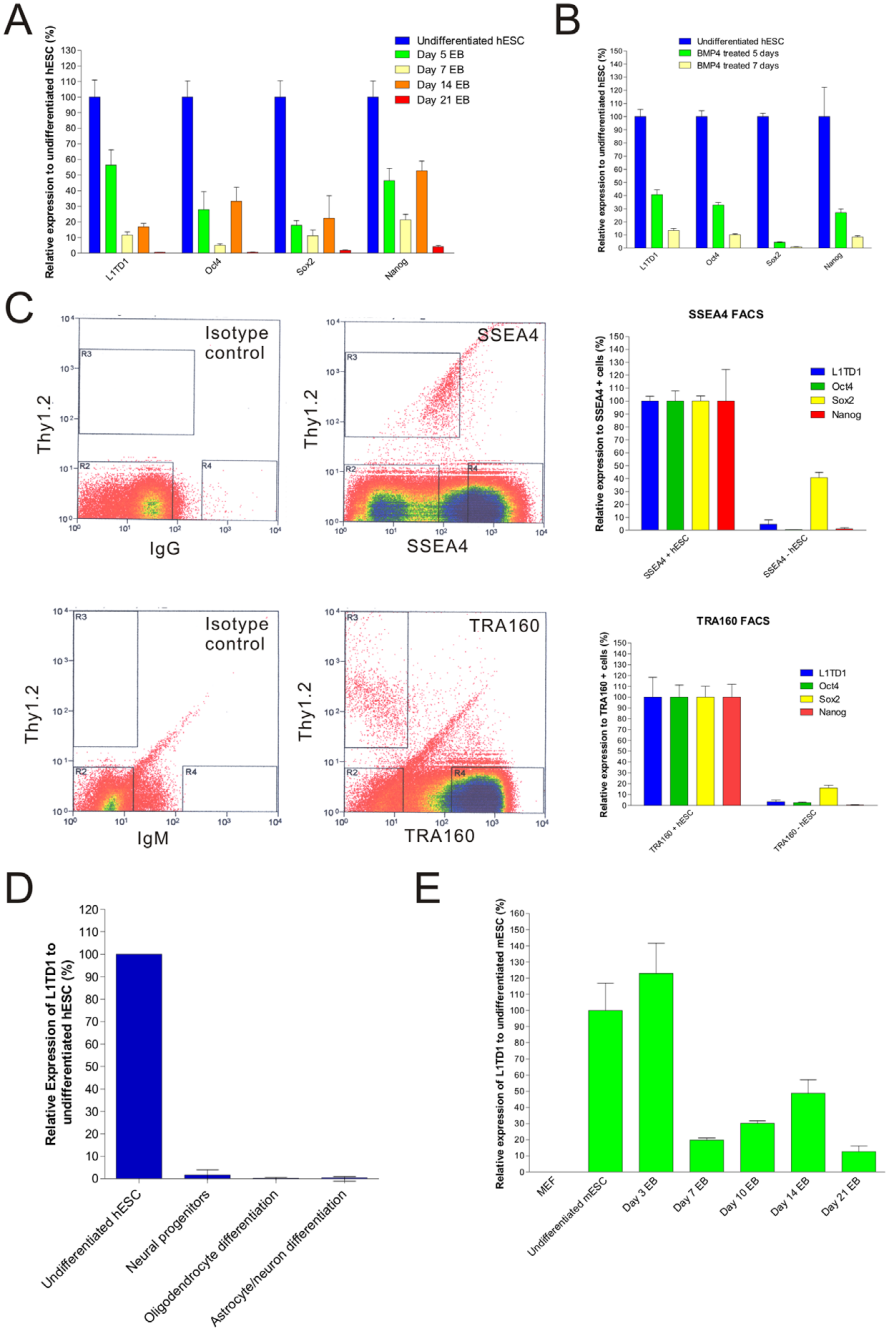
mRNA expression of L1TD1 in undifferentiated and differentiated hESC. Quantitative PCR of L1TD1, Oct4, Sox2 and Nanog expression in undifferentiated hESC compared to A) EB or B) hESC treated with BMP4 at different time points. Average relative expression levels and standard deviations from three quantitative PCR reactions for each sample are shown. Representative results from H9 are shown. C) Flow cytogram of hESC sorted with TRA160 or SSEA4. Negative isotype controls were used to set the gating for Thy1.2, TRA160 or SSEA4. Quantitative PCR of L1TD1, Oct4, Sox2 and Nanog expression was carried out comparing SSEA4+ hESC with SSEA4− hESC (Top panel), or TRA160+ hESC with TRA160− hESC (bottom panel). Average relative expression levels and standard deviations from three quantitative PCR reactions for each sample are shown. Representative results from H9 are shown. D) Quantitative PCR of L1TD1 expression in undifferentiated hESC, neural progenitors, and cells promoted to differentiate into oligodendrocytes, astrocytes and neurons (n = 2). E) Quantitative PCR of L1TD1 expression in MEF, undifferentiated mESC and EB at different time points. Average relative expression levels and standard deviations from three quantitative PCR reactions for each sample are shown. Representative results from R1 are shown and biological repeats were carried out in GSI1 mESC lines (Data not shown).

Using quantitative PCR, we compared the mRNA expression levels of L1TD1 in undifferentiated and differentiated hESC. hESC were differentiated by two methods: differentiation by embryoid body (EB) formation, or differentiation by BMP4 treatment [12]. Our results demonstrate that L1TD1 mRNA is rapidly downregulated upon EB formation (Figure 1A). In day 5 EB, L1TD1 expression is downregulated to 56±17% of the level in undifferentiated hESC. By day 21, L1TD1 mRNA is almost undetectable (0.4% of the level in undifferentiated hESC). This expression pattern of L1TD1 observed upon EB differentiation is similar to other known pluripotent markers including Oct4, Sox2 and Nanog (Figure 1A). Similar results were observed with mESC, in which EB differentiation also led to downregulation of L1TD1 transcripts (Figure 1E). Notably, mouse embryonic fibroblast (MEF) feeders do not express the L1TD1 gene (Figure 1E). Next, we differentiated hESC using BMP4 treatment. This also resulted in downregulation of L1TD1 mRNA, in a trend similar to the downregulation of Oct4, Sox2 and Nanog (Figure 1B). hESC treated with BMP4 for 5 days displayed 41±7% of the level of L1TD1 expression in comparison to untreated hESC. By day 7 following BMP4 treatment, the level of L1TD1 dropped down to only 13±3% compared to untreated hESC (Figure 1B). Therefore our data suggests that L1TD1 mRNA is rapidly downregulated upon hESC differentiation. Similar results are observed in another cell line H1 (Figure S1A).

Since residual undifferentiated hESC may remain even after prolonged differentiation following EB formation or BMP4 treatment, this may introduce undesired variability in our quantitative PCR results. To address this issue of heterogeneity, we used fluorescence-activated cell sorting (FACS) to separate undifferentiated and differentiated hESC populations using the known stem cell markers TRA160 and SSEA4. In addition, we excluded the MEF feeders from our sample using antibodies to the MEF marker Thy1.2 [13]. As illustrated in Figure 1C, our results showed that L1TD1 mRNA is downregulated in SSEA4-negative hESC (5±3% of SSEA4 positive-hESC) or TRA160-negative hESC (3±2% of TRA160-positive hESC). An analysis of Oct4, Sox2 or Nanog mRNA levels in SSEA4-negative or TRA160-negative hESC showed similar downregulation of these factors as expected. Similar results were obtained for hESC sorted with TRA181, an antibody that recognizes different epitopes of the same antigen as TRA160 (data not shown). We have also confirmed the results in two different hESC line, H9 (Figure 1C) and H1 (Figure S1B), supporting the notion that L1TD1 can be used to identify undifferentiated hESC in multiple cell lines.

Since a small portion of the SSEA4-negative or TRA160-negative hESC retain expression of L1TD1 (Figure 1C), we sought to determine what lineage of differentiated hESC tends to retain L1TD1. In Figure 1D, we showed that hESC promoted to differentiate along the neural lineage do not express L1TD1, as we failed to detect L1TD1 expression in hESC-derived neural progenitor, and cells promoted to differentiate into oligodendrocytes, astrocytes and neurons. *In silico* analysis of L1TD1 expression using the EST profile viewer from the Unigene (http://www.ncbi.nlm.nih.gov/unigene) revealed that L1TD1 is absent in many adult tissues, except for low expression level in blood tissues, connective tissues, placenta and the testis (Figure S2). Thus, we cannot rule out the possibility that hESC differentiated into these tissues may retain low expression of L1TD1.

**Figure 2:**
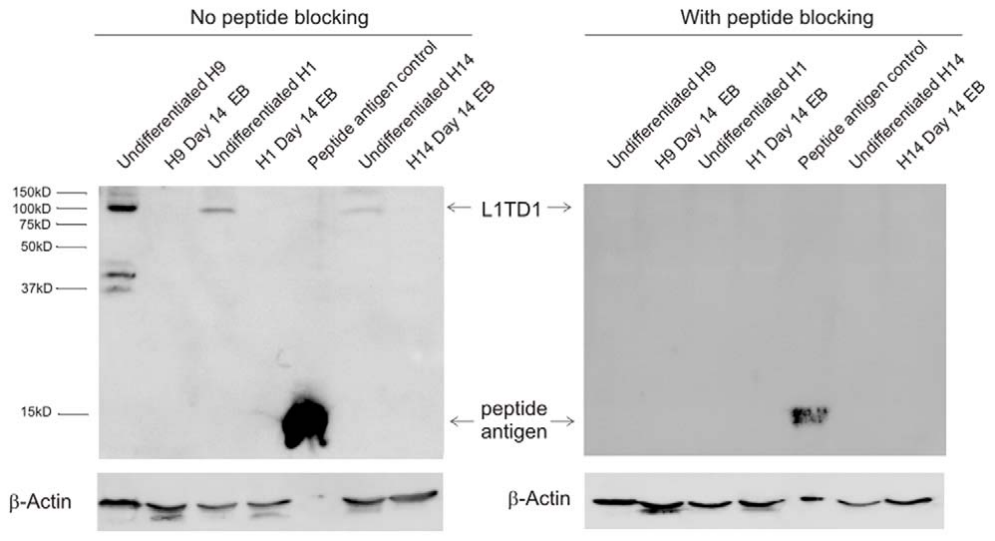
Protein expression of L1TD1 in undifferentiated and differentiated hESC. Left) Western blot analysis of L1TD1 in undifferentiated H1, H9 and H14 cells and the corresponding Day 14 EB. 2 µg of peptide antigen for generation of the L1TD1 antibody was used as a positive control. Right) Peptide blocking of the L1TD1 antibody was performed to ensure the specificity of the L1TD1 antibody.

To study the protein expression of L1TD1 in hESC, we generated an anti-L1TD1 antibody in rabbits against a synthetic peptide corresponding to the C-terminal sequence of human L1TD1 (amino acids 686–699). Using western blot analysis, L1TD1 protein is detected as a ∼100 kDa band in undifferentiated hESC (Figure 2), similar to the expected molecular size of L1TD1 calculated base on its amino acid sequence (∼98 kDa). However, such band is not detectable in Day 14 EB (Figure 2). Two other bands (∼37 kDa and ∼40 kDa) are also detected in some experiments but it is not consistently detected. Furthermore, pre-incubation of the anti-L1TD1 antibody with the peptide antigen eliminated all bands, suggesting that the anti-L1TD1 antibody is specific to the designed sequence of human L1TD1 gene (Figure 2). However, this anti-L1TD1 antibody did not work for immunocytochemistry (data not shown). Nevertheless, our results suggest that L1TD1 protein is abundant in undifferentiated hESC and downregulated upon differentiation, rendering it a useful marker for undifferentiated hESC.

### L1TD1 is a downstream target for Nanog

**Figure 3:**
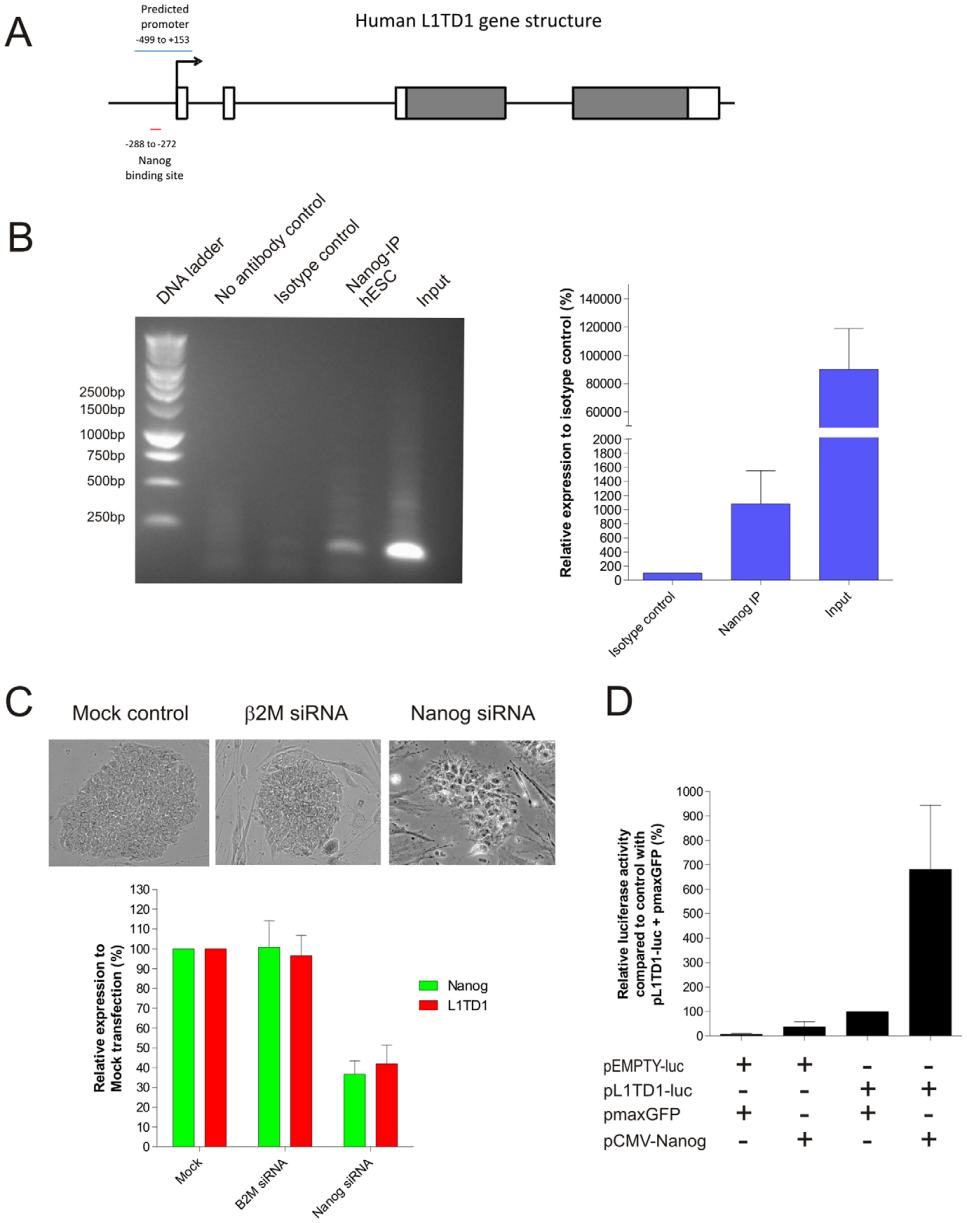
L1TD1 is a downstream target of Nanog. A) Schematic diagram of the L1TD1 gene structure showing the presence of a Nanog binding site in the predicted promoter region. Exons are depicted as boxes and the coding region of L1TD1 are shaded. B) ChIP results of H9 sample immunoprecipitated with antibody to Nanog. Negative controls were performed with samples immunoprecipitated in the absence of antibody or with an isotype-matched antibody. The input is a positive control of samples prior to immunoprecipitation. The picture is a representative result from three independent experiments. Quantitative PCR is performed to quantify the ChIP samples. Average relative expression levels and standard deviations from three independent experiments are shown. C) Morphology of hESC nucleofected with siRNA against Nanog, ß2M and a mock control (Top panel). Quantitative PCR of L1TD1 and Nanog expression in hESC nucleofected with siRNA against Nanog, ß2M and a mock control (Bottom panel). Average relative expression levels and standard deviations from three independent experiments are shown. D) Luciferase assay of SW480 co-transfected with different combination of the following vectors: a promoterless luciferase reporter vector (pEMPTY-luc), a luciferease reporter vector driven by the L1TD1 upstream promoter region (pL1TD1-luc), an overexpression vector for GFP (pmaxGFP) or Nanog (pCMV-Nanog). Average relative luciferase activity and standard deviations from three independent experiments are shown.

Using the Genomatix program (http://www.genomatix.de), we identified a Nanog binding site (AATG) ∼280 bp upstream of the transcription start site in the predicted promoter region of L1TD1 (Figure 3A). Therefore, we analyzed the association of Nanog to the L1TD1 gene in undifferentiated hESC by chromatin immunoprecipitation (ChIP). Using quantitative PCR, our results demonstrated a 10.8-fold enrichment of Nanog association to the upstream region of L1TD1 in undifferentiated hESC compared to the isotype-matched control, suggesting L1TD1 is a downstream target of Nanog (Figure 3B). No band is observed in our negative controls using samples immunoprecipitated in the absence of antibody or with an isotype antibody, indicating the immunoprecipitation procedure of our ChIP assay is specific. Moreover, we carried out siRNA-mediated knockdown of Nanog in hESC. As shown in Figure 3C, knockdown of Nanog expression levels (37±12% of mock transfected hESC) resulted in a differentiated morphology in hESC, as well as downregulation of L1TD1 transcripts (42±16% of mock transfected hESC). We also showed that knockdown of a control gene ß2M has no effect on the expression of L1TD1 (Figure 3C).

To further study the interaction between Nanog and the L1TD1 gene, we performed luciferase assay by constructing a luciferase reporter vector driven by the upstream promoter region of L1TD1 (pL1TD1-luc). As hESC possess high level of endogenous Nanog expression, we opted to perform the luciferase assay in SW480 cells, a human colon adenocarcinoma cell line without detectable level of endogenous Nanog expression (data not shown, and [14]), in order to minimize background noise of luciferase reading. Our luciferase assay results demonstrated that ectopic expression of Nanog activates the L1TD1 upstream promoter region, resulting in a 6.8 fold increase in luciferase activity compared to control (Figure 3D). We verified that this increase in luciferase activity is specific to the interaction between Nanog and the L1TD1 upstream promoter region, as we observed low luciferase activity in our negative controls without the L1TD1 upstream promoter region or with ectopic expression of GFP (Figure 3D). Altogether, our results from luciferase assays are consistent with our ChIP and Nanog knockdown studies, providing evidence that L1TD1 is a downstream target of Nanog.

## Discussion

Here we have characterized the expression of a novel gene L1TD1 in hESC. Using quantitative PCR and western blot analysis, we demonstrated that L1TD1 are highly expressed in hESC and rapidly downregulated upon differentiation of hESC by EB formation (Figure 1A and 2) or BMP4 treatment (Figure 1B). Furthermore, L1TD1 expression is downregulated in differentiated SSEA4-negative or TRA160-negative hESC (Figure 1C and Figure S1B). This result is consistent with a previous DNA microarray study that demonstrated L1TD1 is also enriched in undifferentiated hESC [15]. Furthermore, we provided evidence that the L1TD1 protein expression is also downregulated upon hESC differentiation in multiple hESC cell lines. Therefore, L1TD1 can be used as a marker to distinguish undifferentiated hESC from differentiated progenitors and the MEF feeders (Figure 1 and 2).

Our *in silico* analysis indicated that L1TD1 expression is highly enriched during the blastocyst stage of embryogenesis and absent in many adult tissues in human (Figure S2), an expression pattern much like that observed for Nanog [9]. Therefore, we wondered whether Nanog could regulate L1TD1 in hESC. In this study, we showed that Nanog is associated with a site situated in the upstream predicted promoter region of L1TD1, suggesting that L1TD1 expression may be regulated by Nanog (Figure 3B). Using luciferase reporter assay, we provided evidence suggesting that Nanog can bind to the upstream promoter region of L1TD1 and activate its expression (Figure 3D). Consistent with this idea, we also demonstrated that Nanog knockdown in hESC resulted in downregulation of L1TD1 expression (Figure 3C).

At the moment, no previous study has addressed the functional role of L1TD1. Our *in silico* analysis revealed a full Transposase 22 domain near the C terminus and another truncated Transposase 22 domain in human L1TD1 (data not shown). A previous study has described the Transposase 22 domain as a misnomer and that this domain does not encode for a transposase, but actually represents the open reading frame (ORF) 1 protein of the Long interspersed element-1 (L1) retrotransposon [16], also known as leucine zipper protein p40 [17]. A functional L1 element encodes for two proteins, ORF1 protein and ORF2 protein (reviewed in [18]). Whereas ORF2 encodes for a protein with endonuclease and reverse transcriptase activity [19], [20], [21], less is known about the role of ORF1. Mutagenesis studies indicated that ORF1 is required for L1 retrotransposition [21], [22]. In this aspect, a previous study found that undifferentiated hESC can support L1 retrotransposition *in vitro* [23], thus L1TD1 may play a role in regulating L1 retrotransposition in hESC. Also, the ORF1 protein possess RNA-binding [24], [25] and RNA chaperone activity [26]. Therefore, it is possible that L1TD1 may exhibit similar functions as well. Moreover, a yeast-two-hybrid screen revealed that ORF1 protein can bind to components of the RNA-induced silencing (RISC) complex involved in RNA interference, as well as other proteins involved in mRNA transport [27]. Given that L1TD1 protein contain the Transposase 22 domain/L1 ORF1, it is possible that L1TD1 can bind to the same binding partners as L1 ORF1. Clearly, further research is needed to evaluate the precise function of L1TD1 in hESC. In summary, we have demonstrated that the novel gene L1TD1 can be used as a marker for undifferentiated hESC and is a downstream target of Nanog.

## Materials and Methods

### Cell culture

hESC cell lines H1, H9 and H14 (WiCell) were cultured on mitotically-inactivated MEF (Millipore) and media supplemented with 20% knockout serum replacement and 4 ng/ml bFGF (Invitrogen) as described by Xu *et al.* (2001) [28]. Medium was changed every day and cells were passaged using 1 mg/ml collagenase IV (Invitrogen) every 7 days. hESC were maintained in an incubator at 37°C with 5% CO_2_. In some experiments, hESC were treated with 25 ng/ml of BMP4 (R&D Systems) to induce differentiation as described by Pera *et al.* [12]. EB were formed by growing hESC in suspension in a low attachment culture plate with media supplemented with 20% knockout serum replacement. H9 hESC-derived neural progenitor cells (Millipore) were maintained in ENStem-A neural expansion medium (Millipore) on plates pre-coated with poly-l-ornithine (Sigma) and laminin (Sigma), according to manufacturer's instructions. hESC-derived neural progenitor cells were differentiated into oligodendrocytes, neurons and astrocytes with protocols described in [29]. Briefly, hESC-derived neural progenitor cells were cultured in DMEM supplemented with N1 (Invitrogen) and PDGF-A (2 ng/ml, Peprotech) for 8 days to promote oligodendrocyte differentiation. Alternatively, the neural progenitor cells were promoted to differentiate into neurons and astrocytes by culturing on ornithine/laminin substrate for 8 days with medium consisting of DMEM/F12, N2 supplement (Invitrogen), cAMP (100 ng/ml, Sigma), and brain-derived neurotrophic factor (BDNF, 10 ng/ml; PeproTech). mESC cell lines R1 and GSI1 were cultured in media containing leukemia inhibitory factor (LIF, Millipore, 1000Units/ml) and 16% fetal calf serum (Hyclone). R1 mESC were cultured in feeder-free conditions whereas GSI1 mESC were cultured in the presence of MEF. EB were formed by growing mESC in suspension in low attachment culture plates in media without LIF. SW480 cells were maintained in DMEM supplemented with 10% fetal calf serum (Hyclone), 1% L-glutamine and 0.5% penicillin/streptomycin (All from Invitrogen).

### Quantitative PCR

RNA samples were extracted from H1, H9 and H14 cells using an RNeasy kit (Qiagen). cDNA was synthesized from 1 µg RNA using the high capacity cDNA reverse transcription kit (Applied Biosystems). Taqman mastermix and probes (Oct4: Hs00999632_g1; Sox2: Hs01053049_s1; Nanog: Hs02387400_g1; L1TD1: Hs00219458_m1; 18S: Hs99999901_s1) were all purchased from Applied Biosystems. Quantitative PCR was performed following the manufacturer's instructions. Samples were analyzed in a 7900HT qPCR machine (Applied biosystems). Briefly, samples were run in a 384 well plates with the following thermal profile: 95°C for 10 minutes, followed by 40 cycles of 95°C for 15 seconds and 60°C for 1 minute. The Ct threshold was set using the parameters by the SDS software (Applied biosystems) and subsequently confirmed manually to obtain the appropriate Ct value. The results were normalised to the housekeeping gene 18S and analysed using the ΔΔCt method as described by Bookout *et al.* [30].

### Fluorescence-activated cell sorting (FACS)

H1 and H9 cells were harvested and dissociated with 0.25% trypsin/EDTA (Invitrogen). The samples were blocked in 10% serum and immunostained with TRA160, TRA181 (Santa Cruz) or SSEA4 (Caltag laboratories) followed by an Alexa-fluor 488 secondary antibody (Invitrogen). Finally, the samples were stained with the PE-conjugated Thy1.2 antibody (BD Pharmingen) to allow sorting of the MEF from hESC [13]. Samples were sorted using a flow cytometer (MoFlo). Isotype-matched controls were used to set the gating for FACS sorting.

### Antibody generation

A polyclonal antibody was generated in rabbits against a synthetic peptide corresponding to amino acids 686–699 of human L1TD1 (SKERQRDIEERSRS) (Genescript). An N-terminal cysteine was added to the peptide to allow it to conjugate with Keyhole Limpet Hemocyanin (KLH). Two separate rabbits were immunized with the peptide antibody on day 1, 14, 35 and 56. Anti-sera were collected 14 days after the final immunisation. The antiserum was affinity-purified using the SulfoLink immobilization kit following manufacturer's instructions (Thermo Scientific). Briefly, the peptide antigen is coupled with Sulfolink resin to make an affinity column. Ten ml of antiserum was repeatedly run through the column, and the purified antibodies were eluted in buffer containing sodium azide.

### Western blot analysis

H1, H9 and H14 cells were lysed in Radioimmunoprecipitation (RIPA) buffer and sample reducing buffer containing ß-mercaptoethanol as described previously [31]. Briefly, samples were run in a 4% stacking gel and 10% resolving gel and transferred to a PVDF membrane (GE Healthcare). The membrane was blotted with anti-L1TD1 antibody (1∶2000) followed by incubation with a goat anti-rabbit horseradish peroxide (HRP)-conjugated secondary antibody. Peptide blocking is performed by pre-incubation of the anti-L1TD1 antibody with the synthetic peptide antigen (60 µg) for 2 to 4 hours at room temperature or overnight at 4°C prior to immunoblotting. Chemilluminescent detection reagent (ECL plus, GE Healthcare) was used to detect the HRP signal on film or using the Gel-doc system (Biorad). Subsequently, the membrane was stripped with the Restore western blot stripping buffer (Thermo scientific) and re-blotted with an anti-ß-actin antibody (Santa Cruz) and the appropriate HRP secondary antibody.

### Chromatin immunoprecipitation (ChIP)

ChIP assays with H9 cells were carried out as described previously by Zeng *et al.* [32]. Briefly, 6×10^6^ cells were crosslinked with 1% formaldehyde at 37°C for 10 minutes. The formaldehyde was neutralized by the addition of 125 mM glycine and the cells were lysed with SDS lysis buffer. The nuclear extracts containing DNA fragments of about 500 base pairs were pre-cleared with protein A sepharose beads (GE Healthcare) previously incubated with 1 mg/ml of bovine serum albumin (BSA, Sigma) and 0.2 mg/ml of salmon sperm DNA (Ambion) at 4°C for 20 minutes. Four to eight micrograms of anti-Nanog polyclonal antibody (R&D Systems) were then incubated with the nuclear extracts at 4°C overnight and precipitated with protein A sepharose beads (GE Healthcare). The complex was washed and eluted with 1% SDS and 100 mM NaHCO_3_. Crosslinks were reversed by incubating at 65°C for four to six hours and the DNA was recovered using a gel cleanup kit (Eppendorf). PCR was performed with the Taq PCR core kit (Qiagen) using primers targeting the Nanog binding site upstream of L1TD1 (Forward: CAGTCGTCCAGGTGAGAGAC; Reverse: CCACTAAAGCGCCTCATCAC; annealing temperature 51°C). PCR products were electrophoresed in a 1% agarose gel and imaged using the Gel-Doc Imager (Bio-Rad). Alternatively, quantitative PCR is performed using Sybr green mastermix (Applied biosystems) with the same primer set. A standard curve using cDNA with 5 fold dilutions was constructed to ensure high PCR efficiency and a dissociation curve is constructed to ensure specific target amplification. Quantitative PCR was performed in triplicate in 384 well plates using thermal profile specified by the manufacturer. Samples were analyzed in a 7900HT qPCR machine (Applied biosystems). The results were analysed using the ΔΔCt method as described by Bookout *et al.* [30].

### siRNA-mediated knockdown of Nanog

Nanog knockdown in H9 cells was carried out using nucleofection, following procedures described in [4], [6]. Briefly, H9 cells were trypsinized and nucleofected with 100 nM of siRNA targeting Nanog (AAGGGTTAAGCTGTAACATAC). Cells nucleofected in the absence of siRNA or with 100 nM of siRNA targeting Beta-2-microglobulin (ß2M) were used as negative controls (AAGATTCAGGTTTACTCACGT). Following nucleofection, the cells were plated on MEF and cultured in the presence of neurotrophins (50 ng/ml each of BDNP, NT3 and NT4). RNA samples were harvested 4 days after nucleofection and knockdown of Nanog expression was confirmed by quantitative PCR.

### Luciferase reporter assay

Luciferase reporter plasmid driven by the L1TD1 upstream promoter region (pL1TD1-luc) is constructed by inserting the L1TD1 upstream promoter region (−1230 To +3) into the promoterless pGL4.10 plasmid (Promega). The L1TD1 upstream region is cloned using the following primers: Forward primer: 5′-GGGGAGTTTGGCTCCTGTAGA-3′; Reverse primer: 5′-AAGGACTGAGAGGATTCCCGATC-3′. A promoterless pGL4.10 plasmid (pEMPTY-luc) is used as a negative control to determine background luciferase activity. The plasmid pCMV-Nanog (Origene) is used to overexpress Nanog, alternatively pmaxGFP (Lonza) is used to express GFP as stuffer DNA to gauge transfection efficiency. SW480 were cultured in 12-well plates and co-transfected with the corresponding luciferase reporter vector (100 ng/well) in the presence of pCMV-Nanog or pmaxGFP (1 µg/well) using Express-in (Open biosystems). Cells were harvested 24 hours post-transfection and assay for luciferase activity using the Luciferase reporter assay system (Promega) following manufacturer's instructions. Samples were measured using a luminometer (Berthold Technologies). The luciferase activities were then normalized to cell number by quantification of the total protein concentration in the samples using the Non-interfering protein assay (GBiosciences) following manufacturer's instructions.

## Supporting Information

Figure S1 **L1TD1 mRNA downregulation upon hESC differentiation is observed in different cell line.** A) Quantitative PCR of L1TD1 expression in undifferentiated H1 compared to EB at different time points. Average relative expression levels and standard deviations from three quantitative PCR reactions for each sample are shown. B) Quantitative PCR of L1TD1 expression in H1 sorted with TRA160 or SSEA4. Average relative expression levels and standard deviations from three quantitative PCR reactions for each sample are shown.
(TIF)

Figure S2 ***In silico*** **study of expression profile of L1TD1 in different tissues.** The expression profile of L1TD1 in different tissue samples was extracted from the Unigene database. The abundance of L1TD1 transcripts in different tissue samples are presented as the number of EST transcripts per million (TPM), a value that normalized the L1TD1 EST counts to the total EST counts of the sample.
(TIF)
