# Supplementary figures and images for: L1TD1 Is a Marker for Undifferentiated Human Embryonic Stem Cells
Source: PLoS One. 2011 Apr 29;6(4):e19355. doi: 10.1371/journal.pone.0019355 (PMC3084827; doi:10.1371/journal.pone.0019355)

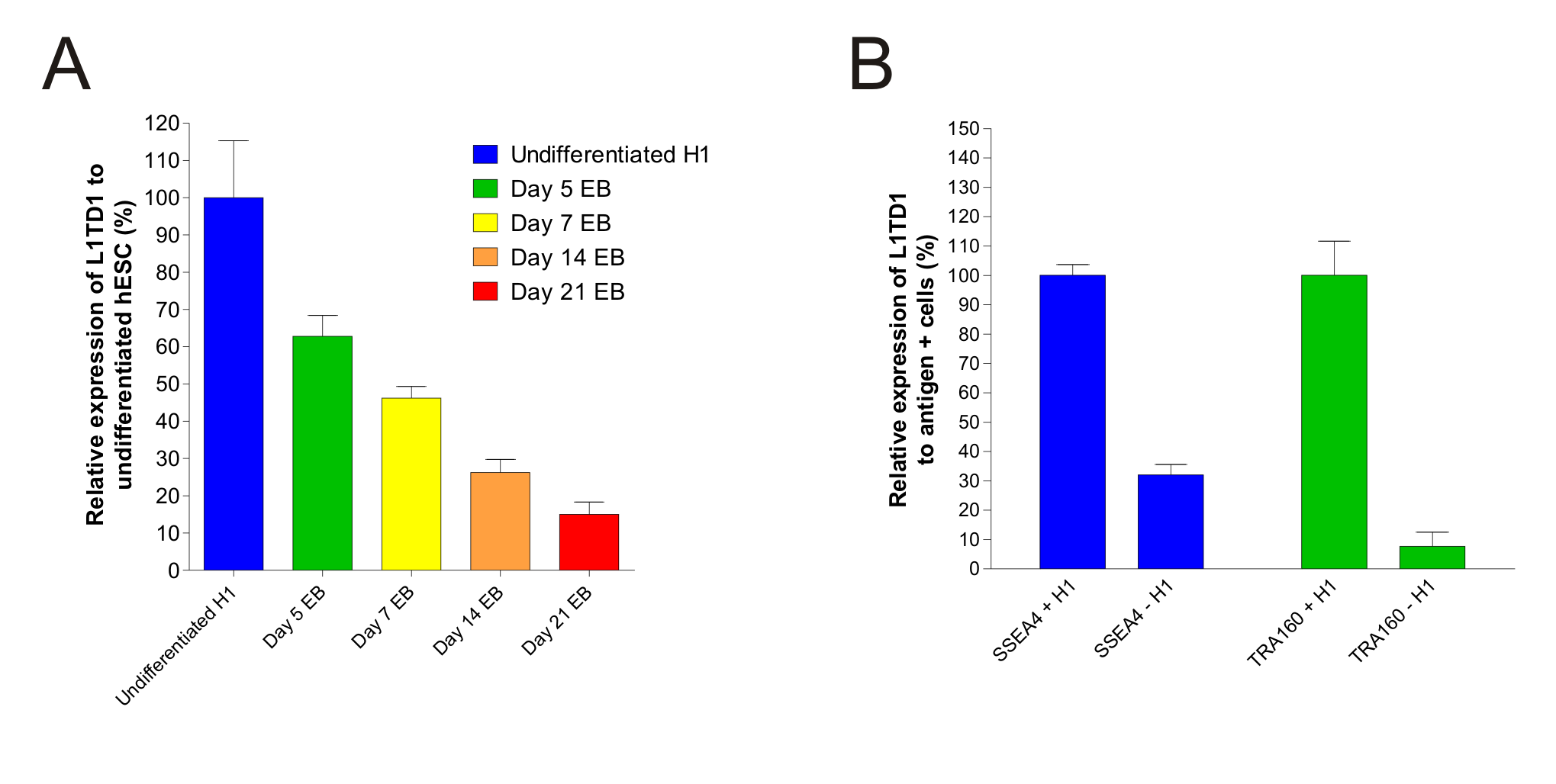

Supplement: Figure S1 — L1TD1 mRNA downregulation upon hESC differentiation is observed in different cell line. A) Quantitative PCR of L1TD1 expression in undifferentiated H1 compared to EB at different time points. Average relative expression levels and standard deviations from three quantitative PCR reactions for each sample are shown. B) Quantitative PCR of L1TD1 expression in H1 sorted with TRA160 or SSEA4. Average relative expression levels and standard deviations from three quantitative PCR reactions for each sample are shown. (TIF) [file pone.0019355.s001.tif]

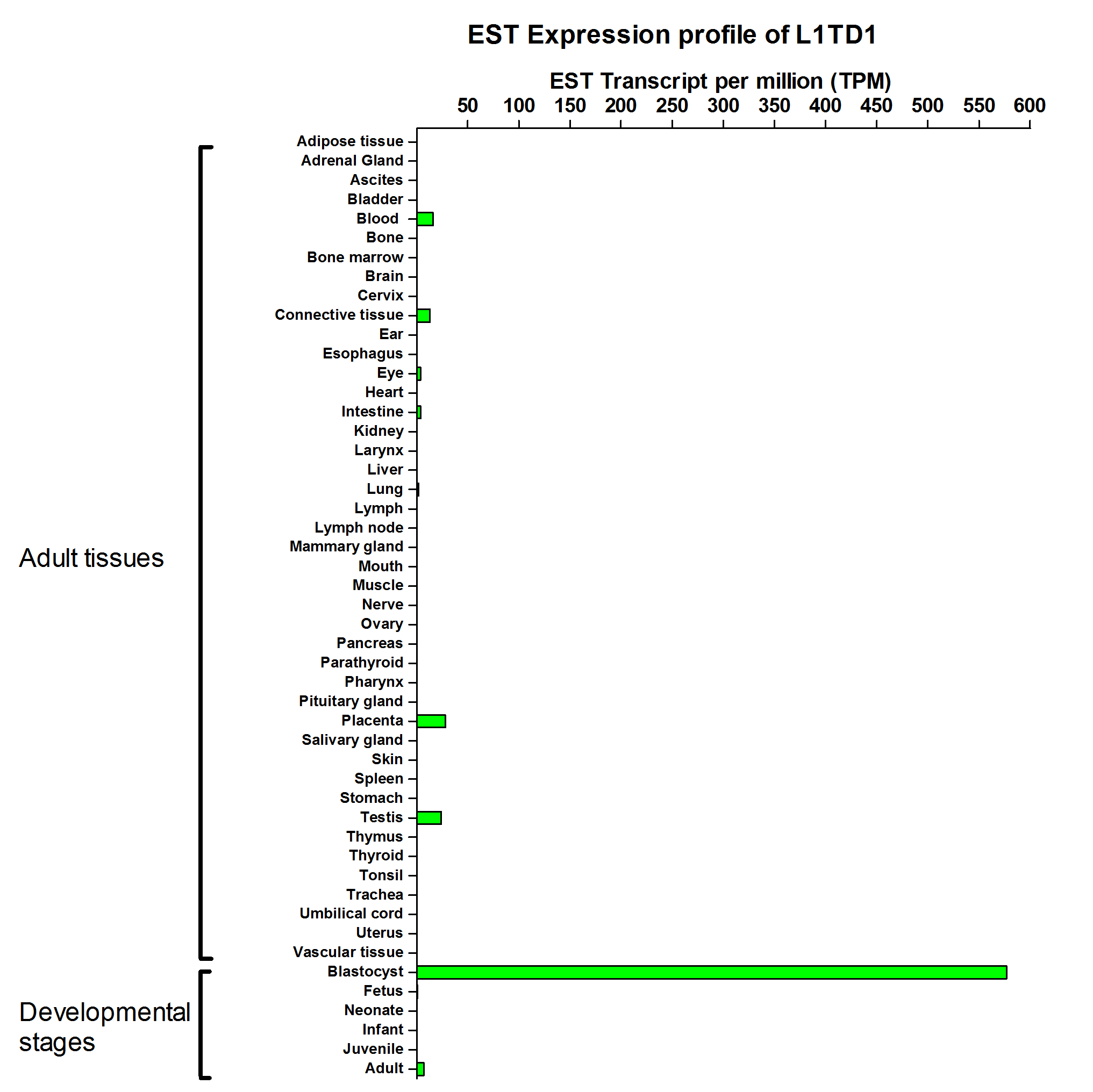

Supplement: Figure S2 — In silico study of expression profile of L1TD1 in different tissues. The expression profile of L1TD1 in different tissue samples was extracted from the Unigene database. The abundance of L1TD1 transcripts in different tissue samples are presented as the number of EST transcripts per million (TPM), a value that normalized the L1TD1 EST counts to the total EST counts of the sample. (TIF) [file pone.0019355.s002.tif]
